# P-575. Evaluation of Weight Gain on Incident Hypertension among People Living with HIV-1 Receiving Dolutegravir (DTG)-Based Regimens or Comparator Antiretroviral Therapy (cART) in Randomized Clinical Trials through 96 Weeks

**DOI:** 10.1093/ofid/ofae631.773

**Published:** 2025-01-29

**Authors:** Clifford B Jones, Sofia Pozsonyiova, Richard Grove, Michael McKenna, Parul Patel

**Affiliations:** ViiV Healthcare, Brentford, UK, London, England, United Kingdom; GSK, Collegeville, Pennsylvania; GlaxoSmithKline, Brentford, UK, Brentford, England, United Kingdom; GSK, Collegeville, Pennsylvania; ViiV Healthcare, Durham, North Carolina

## Abstract

**Background:**

Weight gain has been observed with integrase strand transfer inhibitor (INSTI)-based treatment with an association between INSTI use and hypertension (HTN) in some studies. We observed no difference in odds of incident HTN and minimal weight increase in participants receiving DTG-based or comparator antiretroviral therapy (cART) through Week (W)96. Here, we evaluated the relationship between ART use, weight gain, and HTN risk among participants without baseline (BL) HTN receiving DTG or cART in pooled phase 2/3 studies.

**Methods:**

Data from ART-naive participants randomized to either DTG-based ART (+ ABC/3TC or TDF/FTC), or cART (EFV/TDF/FTC, RAL + 2 NRTIs, or DRV/r + 2 NRTIs), were pooled from the SPRING-1 and -2, SINGLE, and FLAMINGO trials. HTN at BL, W24, W48, and W96 was defined as a single systolic blood pressure (SBP) ≥ 140 mmHg and/or diastolic blood pressure (DBP) ≥ 90 mmHg, HTN history or HTN adverse event, and/or antihypertensive use. Mixed-models repeated-measures (MMRM) analyses evaluated adjusted change from BL in weight. Bayesian Joint Models (JM) evaluated the associations between treatment, weight change, and treatment and weight change interaction with HTN risk. Models were adjusted for key BL variables and pooled treatment.

**Results:**

Among 2345 participants, 23% (n=530) were excluded for BL HTN; of the remaining, 927 received DTG-based ART and 888 received cART. At W96, adjusted mean (SE) change in weight was 1.98 (0.242) kg for DTG-based ART vs 1.59 (0.212) kg for cART (treatment difference: 0.39 kg; 95% CI: −0.25, 1.04; P=0.230). From JM, treatment (DTG-based ART vs cART) was not independently associated with HTN risk through W96 (mean, M [hazard ratio, HR]: 1.099; 95% credible interval [CrI]: 0.876, 1.370). On average, a 1-kg increase in weight was estimated to increase HTN risk (HR) by 1.012 (CrI: 1.004, 1.020). No evidence of treatment separation was observed through the treatment-weight interaction (DTG-based ART HR: 1.013 [1.003, 1.022]; cART HR: 1.009 [0.998, 1.020]).

**Conclusion:**

Weight gain was moderately associated with HTN risk through W96. ART was not an independent risk factor for HTN. Weight gain attributed to treatment effect impacted HTN risk similarly.

**Disclosures:**

**Clifford B. Jones, BSc MSc MB ChB**, GSK: Stocks/Bonds (Public Company)|ViiV Healthcare: Employee **Sofia Pozsonyiova, MS**, GSK: Employee|GSK: Stocks/Bonds (Public Company) **Richard Grove, MSc**, GSK: Employee|GSK: Stocks/Bonds (Public Company) **Michael McKenna, MBChB**, GSK: Employee|GSK: Stocks/Bonds (Public Company) **Parul Patel, MSW**, GSK: Stocks/Bonds (Public Company)|ViiV Healthcare: Employee

